# Myocardial perfusion and flow reserve in the asynchronous heart: mechanistic insight from a computational model

**DOI:** 10.1152/japplphysiol.00181.2023

**Published:** 2023-07-13

**Authors:** Anneloes G. Munneke, Joost Lumens, Theo Arts, Frits W. Prinzen, Tammo Delhaas

**Affiliations:** ^1^Department of Biomedical Engineering, CARIM School for Cardiovascular Diseases, Maastricht University, Maastricht, The Netherlands; ^2^Department of Physiology, CARIM School for Cardiovascular Diseases, Maastricht University, Maastricht, The Netherlands

**Keywords:** asynchronous ventricular activation, coronary circulation, coronary flow regulation, myocardial oxygen demand and supply, myocardial flow reserve

## Abstract

The tight coupling between myocardial oxygen demand and supply has been recognized for decades, but it remains controversial whether this coupling persists under asynchronous activation, such as during left bundle branch block (LBBB). Furthermore, it is unclear whether the amount of local cardiac wall growth, following longer-lasting asynchronous activation, can explain differences in myocardial perfusion distribution between subjects. For a better understanding of these matters, we built upon our existing modeling framework for cardiac mechanics-to-perfusion coupling by incorporating coronary autoregulation. Regional coronary flow was regulated with a vasodilator signal based on regional demand, as estimated from regional fiber stress-strain area. Volume of left ventricular wall segments was adapted with chronic asynchronous activation toward a homogeneous distribution of myocardial oxygen demand per tissue weight. Modeling results show that *1*) both myocardial oxygen demand and supply are decreased in early activated regions and increased in late-activated regions; *2*) but that regional hyperemic flow remains unaffected; while *3*) regional myocardial flow reserve (the ratio of hyperemic to resting myocardial flow) decreases with increases in absolute regional myocardial oxygen demand as well as with decreases in wall thickness. These findings suggest that septal hypoperfusion in LBBB represents an autoregulatory response to reduced myocardial oxygen demand. Furthermore, oxygen demand-driven remodeling of wall mass can explain asymmetric hypertrophy and the related homogenization of myocardial perfusion and flow reserve. Finally, the inconsistent observations of myocardial perfusion distribution can primarily be explained by the degree of dyssynchrony, the degree of asymmetric hypertrophy, and the imaging modality used.

**NEW & NOTEWORTHY** This versatile modeling framework couples myocardial oxygen demand to oxygen supply and myocardial growth, enabling simulation of resting and hyperemic myocardial flow during acute and chronic asynchronous ventricular activation. Model-based findings suggest that reported inconsistencies in myocardial perfusion and flow reserve responses with asynchronous ventricular activation between patients can primarily be explained by the degree of dyssynchrony and wall mass remodeling, which together determine the heterogeneity in regional oxygen demand and, hence, supply with autoregulation.

## Graphical abstract

**Figure:**
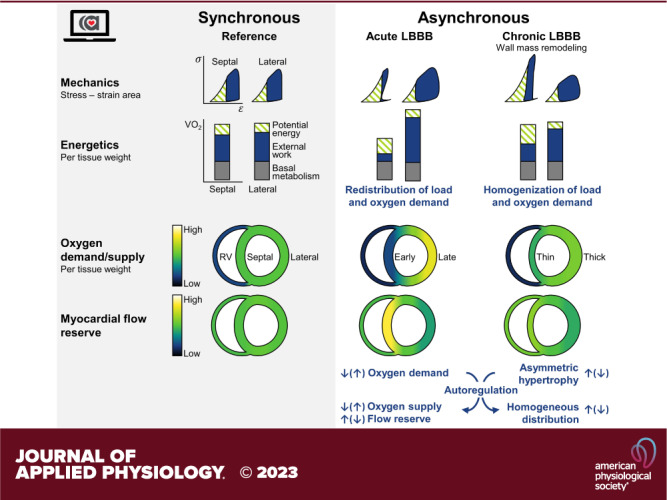

## INTRODUCTION

Asynchronous ventricular activation is associated with regional differences in contraction patterns, stroke work (1, 2), perfusion, and glucose uptake (2, 3), even in the absence of coronary artery disease. In left bundle branch block (LBBB), there is a delay in electrical and mechanical activation of the left ventricular (LV) lateral wall with respect to the septal and the right ventricular (RV) lateral wall. This leads to contraction and shortening of the early activated septum while the later activated, still passive, LV lateral wall is prestretched. As a result of myocardial length-dependent activation (i.e., cellular basis of the Frank–Starling law) (4), the LV lateral wall contracts more forcefully at the expense of septal systolic shortening. Hence, myocardial workload is unevenly distributed, with the septum performing little or even negative stroke work while the LV lateral wall is experiencing a higher workload (1, 2). Furthermore, LBBB has been shown to induce structural remodeling, such as asymmetric hypertrophy with the septum being thinner than the LV lateral wall (5–8).

Although myocardial perfusion is presumed to act in accordance with oxygen demand, conflicting observations on the distribution of perfusion have been reported in patients with LBBB. Some studies show septal hypoperfusion compared with the lateral wall (9–13), whereas others show a homogeneous perfusion (14–17). It remains unclear whether reduced septal perfusion in LBBB, even in absence of coronary artery disease, represents the pathophysiological restriction of flow due to abnormal asynchronous contraction or whether it represents the physiological adaptation to altered myocardial oxygen demand (12, 13). Furthermore, the inconsistency in the observations, although not well understood, may in part be related to the degree of structural remodeling. In particular, a study in canine hearts showed that LV pacing acutely induced a heterogeneous distribution of myocardial perfusion, which was restored after 6 mo of pacing (6). The authors suggested that local myocardial mass was regulated in proportion to metabolic demand (6), thereby equalizing the relative myocardial blood flow distribution.

The aim of the present study was to investigate whether coronary autoregulation can explain the distribution of myocardial perfusion and flow reserve as observed in patients with LBBB and to what extent asymmetric hypertrophy influences this distribution. To this purpose, the multiscale CircAdapt modeling framework for cardiac-to-coronary coupling (18) was built upon by introducing coronary flow regulation to myocardial oxygen demand, enabling investigation of the isolated effect of acute LBBB (dyssynchrony-induced alterations) and chronic LBBB (demand-driven growth-induced alterations) on cardiac mechanics and myocardial oxygen demand, perfusion, and flow reserve. In this modeling framework, regional coronary perfusion is dependent on myocardial contraction through the intramyocardial pressure, which is, in turn, determined by global pump mechanics and regional myofiber mechanics.

## METHODS

### Overview of the Model

**Figure 1.**
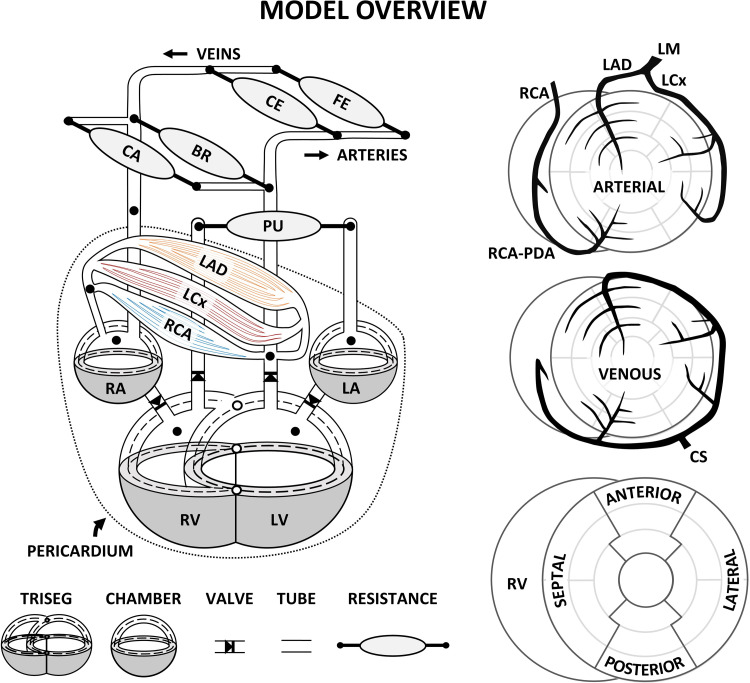
Schematic representation of the closed-loop cardiovascular CircAdapt model, including the TriSeg module (20) [which allows mechanical interaction at the junction (○) between the left ventricle (LV) and right ventricle (RV) and the septal (S) wall], the chamber module (19), valve module (24), one-dimensional tube module (22), and zero-dimensional arteriovenous resistances for systemic and pulmonary circulation (19) as well as coronary circulation (18). The dashed line indicates the midwall volume; the dotted line indicates the pericardium, which surrounds the heart and coronary circulation. The left ventricular and septal wall were divided into 12 and 5 segments, respectively, using the MultiPatch module (21). Coronary territories of the left circumflex coronary artery (LCx), left anterior descending (LAD), and right coronary artery (RCA) were assigned to individual segments according to the American Heart Association (23). BR, brachial; CA, cerebral; CE, celiac; CS, coronary sinus; FE, femoral; LA, left atrium; LM, left main coronary artery; PDA, posterior descending coronary artery; PU, pulmonary; RA, right atrium.

The modeling framework presented in this article builds upon the previously validated CircAdapt modeling framework for cardiac-to-coronary coupling (18) by taking into account demand-driven coronary flow autoregulation. In brief, the CircAdapt modeling framework (18–22) consists of a network of different modules describing myocardial walls, cardiac valves, large blood vessels, as well as the pulmonary, systemic, and coronary circulations (www.circadapt.org). The LV lateral wall, interventricular septum, and RV lateral wall are mechanically coupled in a junction, and ventricular mechanical interaction is established by equilibrium of tensile forces in the junction (20). Coronary territories are assigned to specific myocardial segments as proposed by the American Heart Association (23) by dividing the LV lateral wall and septal wall into 12 and 5 wall segments (21), respectively (Fig. 1). In each myocardial wall segment, the myofiber stress-strain relation is determined by a three-element Hill-type model describing active and passive cardiac myofiber mechanics (19). Global pump mechanics are related to myofiber mechanics in the three ventricular walls, using the principle of conservation of energy.

The coronary module consists of a one-dimensional (1-D) network of 59 major arteries and veins, as well as a lumped parameter model with three transmural layers for each myocardial wall segment within the coronary territories of the left circumflex (LCx), left anterior descending (LAD), and right coronary artery (RCA) (18). The left main coronary artery and RCA stem directly from the ascending aorta and form the inlets of the coronary arterial tree, based on a simplified version of the anatomical data reported by Dodge et al. (25, 26). Distal ends of the coronary venous tree were connected to the coronary sinus, which then drains into the right atrium. The vessels were modeled as 1-D nonlinear elastic tubes with a fixed length and variable cross-sectional area, to account for pulse wave propagation and flow distribution (22).

### Regional Coronary Flow

Regional coronary flow was made to be impeded by myocardial contraction through extravascular forces (often termed intramyocardial pressure, IMP) and was regulated to regional oxygen demand estimated from global pump mechanics and regional myofiber mechanics.

#### Calculation of intramyocardial pressure.

As described in detail in our previous study (18), IMP was assumed to be a summation of two mechanisms, namely, transmission of cavity pressure into the myocardial wall (cavity-induced extracellular pressure; CEP) and the effect of myocardial stiffness (labeled as varying elastance; VE). CEP accounts for the transmural gradient of IMP measured in vivo and was assumed to vary linearly from ventricular cavity pressure (*P*_1_) at endocardium to pericardial pressure (*P*_2_) at epicardium for the LV and RV lateral walls. For the septal wall, CEP varied linearly from LV cavity pressure (*P*_1_) to RV cavity pressure (*P*_2_). VE was assumed to be layer-independent and related to fiber stress (σ_f_).

(*1*) $$IMP=\underset{CEP}{\underbrace{\left(r\cdot P_{1}+(1-r)\cdot P_{2}\right)}}+\underset{VE}{\underbrace{\gamma \sigma_{f}}},$$

where γ is a prescribed scaling parameter chosen to produce a peak VE equal to ∼20% of peak LV pressure (27, 28), and *r* is the radial position of the subepicardial, mid-, and subendocardial layer prescribed at 1/6, 3/6, and 5/6 of the myocardial wall segment, respectively. Note that the same value of γ = 0.06 was chosen for the left, right, and septal wall segments.

#### Calculation of regional oxygen demand.

Myocardial oxygen demand is governed by three principal factors: heart rate, contractility, and wall tension (29). These mechanical determinants are related to myocardial oxygen consumption through the ventricular pressure–volume area (PVA), as shown in an experimental study by Suga (30). The PVA is equal to the sum of external mechanical work performed during systole and end-systolic potential energy. It represents the total mechanical energy generated by ventricular contraction, even during heartbeats with negative external work (31). In analogy to the PVA concept, the fiber stress-strain area (SSA) reflects regional myocardial work and oxygen consumption (32).

**Figure 2.**
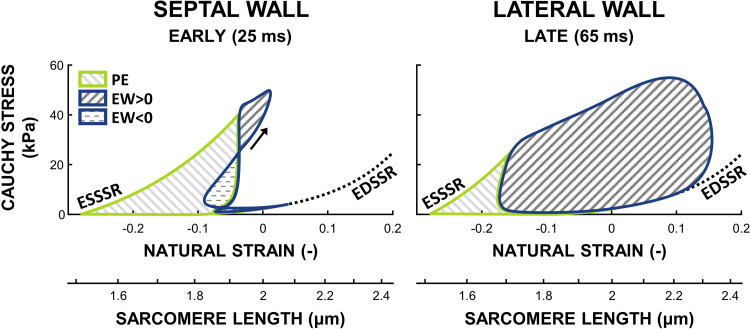
Schematic of the calculation of regional work during asynchronous activation in the early activated septum and late-activated lateral wall. Regional potential energy (PE) was defined as the area enclosed by the end-systolic stress-strain relation (ESSSR), diastolic limb of the stress-strain loop, end-diastolic stress-strain relation (EDSSR), and the *x*-axis. Regional external work (EW) was defined as the area of the stress-strain loop. Note that the stress-strain loop of the early activated septal wall partly exhibits negative external work. Total work is the sum of PE and EW.

Hence, in the model, regional external work was defined as the area enclosed by the Cauchy myofiber stress-natural strain relation (Fig. 2). Regional potential energy was defined as the area enclosed by the end-systolic stress-strain relation, diastolic limb of the stress-strain loop, end-diastolic stress-strain relation, and the *x*-axis, under the assumption that no stress is developed at estimated sarcomere length at zero transmural pressure (32). Total SSA (in Joule) was calculated as the sum of regional external work and potential energy, multiplied by the wall segment’s volume. A more extensive description of SSA calculation can be found in the Supplemental material (see https://doi.org/10.5281/zenodo.8113501).

The relation between SSA and regional oxygen demand (V̇o_2_) was described by a linear relation

(*2*) $${\dot{V}O}_{2}=c_{1}\cdot \frac{\text{SSA}}{t_{cycle}}+c_{2},$$

with *c*_1_ = 4.94 mmol·kJ^−1^ and *c*_2_ = 24.2 mmol·m^−3^·s^−1^ adopted from experimental studies in the paced canine heart (32).

#### Coronary flow regulation.

Coronary flow regulation is understood to involve multiple elements including intramyocardial and perfusion pressure, as well as myogenic, metabolic, endothelial, neural, and hormonal influences (33). Together, these mechanisms regulate coronary flow to ensure adequate oxygen delivery and thus maintenance of normal cardiac function.

Under the assumption that total reference myocardial oxygen demand correlates to total reference coronary flow of 4% of the cardiac output (5.1 L/min), the ratio between current (V̇o_2_) and previous (V̇o_2,old_) myocardial oxygen demand was used to incrementally alter the target coronary flow *q*_0_ as follows

(*3*) $$q_{0,\text{new}}=q_{0,\text{old}}\cdot \frac{{\dot{\text{V}}\text{o}}_{2}}{{\dot{\text{V}}\text{o}}_{2,\text{old}}}.$$

Due to the high myocardial oxygen extraction at rest, increases in myocardial oxygen demand are mediated principally by an increase in coronary blood flow through local vasodilatory response of arterioles (33) and, to a lesser extent, of small arteries (34). Hence, coronary flow regulation is prescribed by a vasodilator signal *fDil* for each arteriolar and small arterial compartment of the intramyocardial model that depends on regional oxygen demand.

(*4*) $$fDil_{new}=fDil_{\text{old}}\cdot e^{\alpha \left(1-\bar{q(t)}/q_{o}\right)},$$

where α < 1 is a damping factor that prevents oscillatory behavior. The ratio between current mean coronary flow $\bar{q(t)}$ and target coronary flow *q*_0_ is used to incrementally alter the vasodilation factor *fDil* until $\bar{q(t)}=q_{0}$. Note that if $\bar{q(t)}<q_{0}$, the exponent is positive and *fDil* increases, promoting vasodilation to meet increased metabolic demand. To ensure an average flow reserve >4.0 while maintaining a global endo-to-epi flow ratio (35, 36), left coronary arterioles were allowed to dilate until a maximum of *fDil* = 3 at the subendocardium with a linear decline to *fDil* = 1.70 at the subepicardium. Maximal *fDil* for the right coronary arterioles was set to 2.40 at the subendocardium and 2.30 at the subepicardium, thereby inducing the same maximal *fDil* at the midwall as in the left coronary arterioles. The vasodilation factor *fDil* for the small arteries was scaled with the maximal *fDil* of the arteriolar compartments of the three intramyocardial layers, with a maximum of *fDil* = 1.4 for all coronaries.

The vasodilator signal *fDil* shifts the nonlinear relation between coronary transmural pressure *p*_trans_ and area *A* (18) to the right with vasodilation and to the left with vasoconstriction as follows

(*5*) ptrans(t)=p0((A(t)+0.5AwfDil·A0+0.5Aw)k/3−1−max(0,(A(t)fDil·A0)0.7·(AwfDil·A0)−0.4−1)2).

The transmural pressure *p*_trans_(*t*) of the intramyocardial vessels is equal to intravascular pressure minus intramyocardial pressure. At reference pressure *p*_0_, the cross-sectional area of the vessel lumen and vessel wall area are equal to *A*_0_ and *A_w_*, respectively. The second term between brackets in *Eq. 5* was added to cope with negative transmural pressures and was based on the collapsible tube law (37, 38). Note that an increase in *fDil* allows for a larger vessel area at similar transmural pressure, analogous to smooth muscle relaxation to decrease vascular tone.

Based on the assumption that resistance of a vessel depends on volume *V* according to Poiseuille’s Law (39), the flow *q*(*t*) across a coronary vessel is assumed to depend on the pressure difference Δ*p*(*t*) between the proximal and distal compartment as follows:

(*6*) $$q\left(t\right)=\left(\frac{q_{0}}{\Delta p_{0}}\cdot \frac{V{\left(t\right)}^{2}}{V_{0}^{2}}\cdot \frac{1}{fDil^{2}}\right)\cdot \Delta p(t),$$

where the reference pressure difference Δ*p*_0_ attributes to the resistance by Ohm’s Law and *V*_0_ refers to the equal distribution of proximal and distal reference volumes. Note that *fDil* compensates for changes in metabolic demand *q*_0_ by changing the vessel’s lumen, implying a change in reference volume *V*_0_. Intuitively, *fDil* can be seen as the required change in resistance to meet metabolic demand $\bar{q(t)}=q_{0}$.

### Simulation Protocol and Data Analysis

#### Simulation of asynchronous ventricular activation.

Starting from a reference simulation representing normal adult cardiovascular mechanics and hemodynamics (18), acute asynchronous ventricular activation was induced as follows. A typical LBBB-like pattern of mechanical activation in the ventricles was modeled by activating the RV lateral wall after a fixed atrioventricular time delay of 139 ms. Earliest mechanical activation of a septal segment occurred 25 ms later. Activation then spreads from segment to segment with a delay of 10 ms, resulting in a total ventricular mechanical activation time of 65 ms.

Heart rate (70 beats/min), cardiac output (5.1 L/min), and mean arterial pressure (92 mmHg) were maintained during all resting simulations by adjusting systemic peripheral resistance and total blood volume. Meanwhile, coronary flow was regulated to myocardial oxygen demand by adjusting the vasodilator signal. All resting simulations shown are at hemodynamic steady state.

#### Simulation of hyperemic flow and reserve.

Hyperemic coronary flow was simulated by increasing the vasodilation factor *fDil* to its maximal value as specified in *Coronary flow regulation*. Total circulating blood volume and systemic peripheral vascular resistance maintained its resting value. Myocardial flow reserve (MFR) was quantified as the ratio of hyperemic to resting flow.

#### Demand-driven remodeling of wall mass.

It has been shown that chronic asynchronous ventricular activation induces a redistribution of myocardial mass: late-activated regions that bear the most load become thicker, whereas early activated regions that perform the least amount of work become thinner (5–8). This goes hand in hand with a more homogeneous myocardial oxygen demand distribution (7). To assess the isolated effect of wall mass remodeling in this study, myocardial oxygen demand was coupled to myocardial growth. Specifically, the wall mass of all LV segments was adapted to homogenize the distribution of myocardial oxygen demand per tissue weight (7), with the maximal allowed change in end-diastolic wall mass set to 25% (3, 6, 7, 40). The maximal value of the vasodilation factor *fDil* was adjusted linearly with the change in wall mass, under the assumption that hyperemic coronary flow is dependent on myocardial mass (40).

### Numerical Implementation

The set of differential equations describing pressure and volume has been solved numerically with the second-order backward differentiation formula with a time step of 1 ms. All simulations were implemented in MATLAB 2019a (The MathWorks, Natick, MA) on a standard personal computer with an Intel CoreTM i7 processor and 16 GB RAM.

## RESULTS

### Coronary Flow and Intramyocardial Pressure Waveforms

#### Reference.

**Figure 3.**
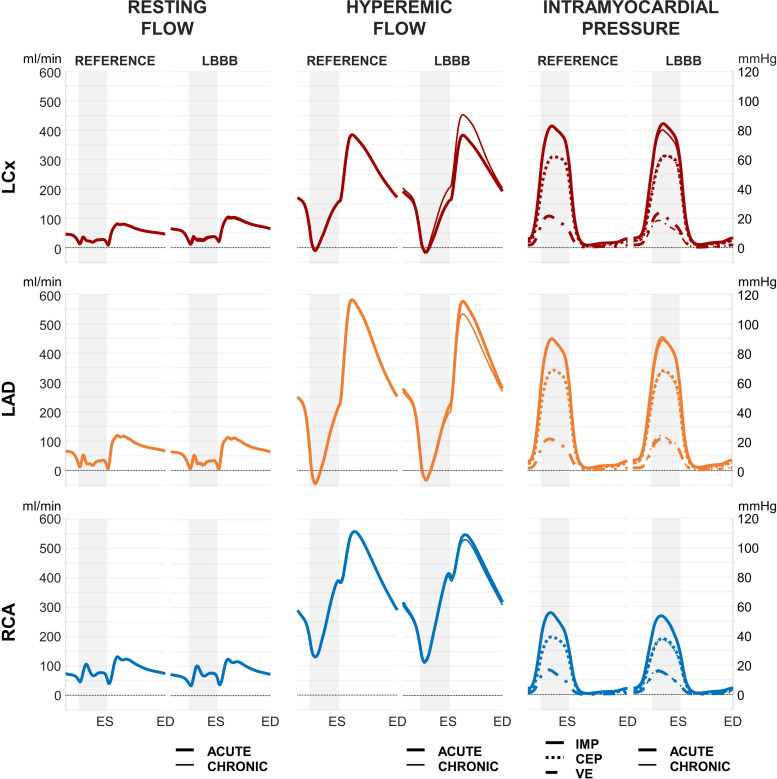
Model-predicted resting and hyperemic flow waveform as well as midwall intramyocardial pressure waveforms for the proximal left circumflex coronary artery (LCx), left anterior descending coronary artery (LAD), and right coronary artery (RCA) with synchronous (REFERENCE) and asynchronous (LBBB) ventricular activation. CEP, cavity-induced extracellular pressure; ED, end-diastole; ES, end-systole; IMP, intramyocardial pressure; LBBB, left bundle branch block; VE, varying elastance.

Figure 3 illustrates the typical biphasic flow waveform in the proximal LAD and LCx with marked diastolic predominance during resting and hyperemic flow conditions, highlighting coronary flow dependence on intramyocardial pressure. The flow waveform of the proximal RCA was more uniformly distributed over the cardiac cycle, mainly due to lower intramyocardial pressure.

#### Acute LBBB.

**Figure 4.**
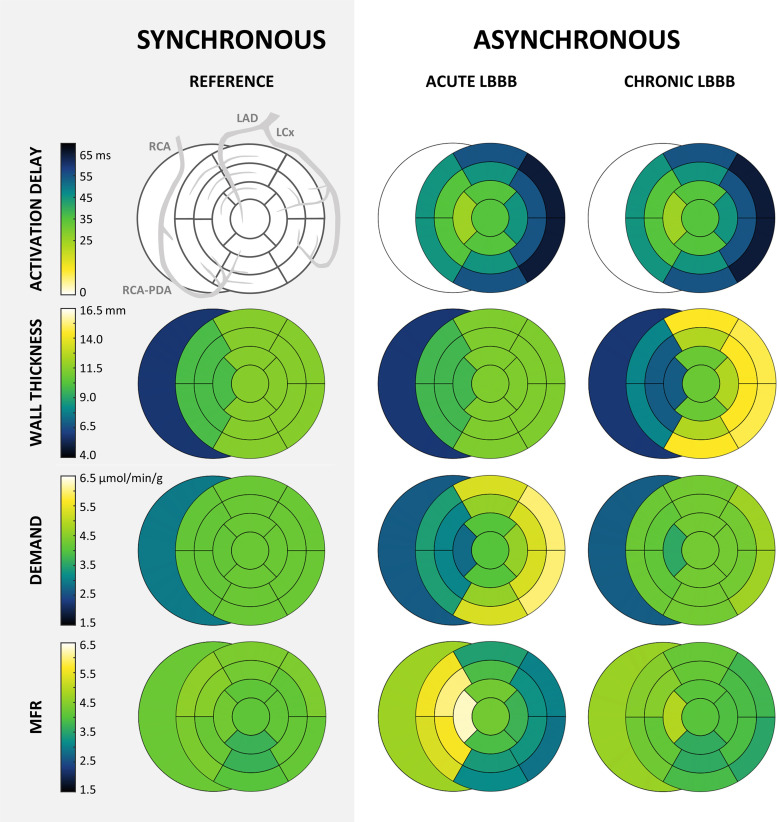
Mapping of activation delay, wall thickness, regional myocardial oxygen demand, and flow reserve under synchronous (REFERENCE) and asynchronous (ACUTE LBBB and CHRONIC LBBB) ventricular activation. LAD, left anterior descending coronary artery; LBBB, left bundle branch block; LCx, left circumflex coronary artery; MFR, myocardial flow reserve; PDA, posterior descending coronary artery; RCA, right coronary artery.

Vasodilation occurred in all LCx sub-branches to meet resting target flow, as is evident from the increased diastolic resting flow in the proximal LCx with acute LBBB compared with the reference. This response was driven by the considerably prolonged isovolumic contraction phase at the expense of diastolic time, by the slightly increased mean IMP (LCx: +5%; LAD: +3%; RCA: +2%; Fig. 3) and by the increased myocardial oxygen demand of approximately +29% (range: 10%–42%; shown in the third row of Fig. 4). In the LAD and RCA territory, however, vasoconstriction occurred in most of the subbranches (5 out 7 and 4 out 6, respectively). The primary factor driving this vasoconstriction was the decrease in average myocardial oxygen demand of 7% (range: −32% to +26%) and 4% (range: −25% to +26%) for the LAD and RCA territories, respectively.

#### Chronic LBBB.

The resting flow in chronic LBBB, compared with acute LBBB remained unchanged since there was no change in absolute myocardial oxygen demand. Mean hyperemic flow in chronic LBBB increased 18% in the proximal LCx and decreased 6% in the proximal LAD compared with acute LBBB, due to small changes in intramyocardial pressure as well as because of its vasodilation dependency on wall mass.

### Myocardial Oxygen Demand and Flow Reserve Distribution

#### Reference.

The distributions of activation delay, wall thickness, myocardial oxygen demand, and MFR are shown as bullseye plots in Figure 4. In addition, bullseye plots of resting and hyperemic myocardial flow can be found in the Supplemental material (https://doi.org/10.5281/zenodo.8113501). A rather homogenous distribution of myocardial oxygen demand and, hence, resting blood flow was found in the reference simulation with synchronous ventricular activation. Differences between the right, septal, and left ventricular walls arise from structural adaptation to hemodynamic loading at 15 L/min in the reference (18) by adjusting cardiac mass, size, and passive stiffness through physiological adaptation rules (41).

MFR was not distributed homogeneously in the synchronously activated heart and decreased toward the apex. Although variations in anteroseptal, anterior, and lateral regions supplied by the LCx and LAD were small [max MFR 4.6 (basal); min MFR: 4.1 (apical)], the posteroseptal and posterior regions supplied by the posterior descending artery (PDA) showed MFR values ranging from 4.4 at the basal segment to 3.7 at the apical segment. This is in line with clinical data showing a considerably lower flow reserve in the PDA compared with the LAD (42).

#### Acute LBBB.

In contrast to synchronous ventricular activation, large regional differences in myocardial oxygen demand and MFR were present during asynchronous ventricular activation. During early systole, the stress-strain loops in early activated regions exhibited a clockwise course, indicating negative work. In contrast, late-activated wall segments demonstrated a significant increase in external work, reaching supranormal values of 194%. Regional potential energy decreased with time of activation. As a result, regional myocardial oxygen demand and blood flow ranged from 68% of its reference value in the early activated septum to 142% of its reference value in the late-activated lateral wall, with a negligible global increase of 2%.

Mean hyperemic flow was nearly identical between synchronous ventricular activation and acute LBBB, differing only 1%. Therefore, MFR decreased in late-activated segments that exhibited an increase in myocardial oxygen demand (LV basal-lateral: 4.3 in the reference vs. 2.9 in acute LBBB) and increased in early activated segments that exhibited a decrease in myocardial oxygen demand (apical-septal: 4.3 in the reference vs. 6.3 in acute LBBB).

#### Chronic LBBB.

Demand-driven remodeling of wall mass helped to equilibrate the myocardial oxygen demand distribution per tissue weight. Note that the change in regional myocardial oxygen demand per tissue weight was considerable compared with the acute LBBB simulation (17% on average), whereas the absolute change in regional myocardial oxygen demand was rather small (2% on average). This demand-driven remodeling induced asymmetric hypertrophy with an increase in lateral wall mass of 21% and an equivalent decrease in septal wall mass of 21%. Consequently, the wall mass-dependent vasodilation and resulting alterations in hyperemic flow, as explained in *Demand-driven remodeling of wall mass*, led to a flow reserve distribution similar to that of the reference.

## DISCUSSION

In the present study, we demonstrated the effects of asynchronous ventricular activation on global and regional myocardial perfusion and reserve. With this model-based approach, we were able to couple myocardial oxygen demand to supply and show how regional myocardial perfusion and reserve are influenced by altered loading conditions and structural remodeling. Our computational findings demonstrate that, in otherwise healthy hearts, LBBB induces a redistribution of absolute myocardial oxygen demand and resting blood flow away from the septum to the LV lateral wall, supporting the hypothesis that septal hypoperfusion is most likely the result of physiological autoregulatory response to reduced septal workload. Because model-predicted regional hyperemic blood flow was unaffected by the time of activation, regional MFR increased with decreases in myocardial oxygen demand and vice versa. Simulated chronic LBBB reproduced clinical findings of asymmetric hypertrophy (5–8) and homogenization of myocardial blood flow per tissue weight (6, 7) and, furthermore, showed restoration of MFR to its reference values. Finally, the observed differences in myocardial perfusion heterogeneity between acute and chronic LBBB may offer a potential explanation for the inconsistent clinical findings regarding myocardial perfusion in asynchronous activation (9–17).

To the best of our knowledge, this is the first modeling framework that considers regional coronary autoregulation to model-predicted myocardial oxygen demand. This is an essential modeling component when studying asynchronous ventricular activation, as mechanical dyssynchrony induces regional changes in cardiac mechanics and myocardial oxygen demand and, hence, in myocardial perfusion and flow reserve.

Although previous studies have focused on constructing models capable of regulating coronary flow (43–46), these models have not specifically addressed the regulation of coronary flow to model-predicted myocardial oxygen demand on a regional level. For instance, in a modeling study by Fan et al. (46) in which ventricular dyssynchrony was prescribed with a time delay in varying elastance of the LCx region compared with the LAD region, simulated regional coronary flow was an imposed modeling result. Instead of regulating flow to varying myocardial oxygen demand, the authors regulated coronary flow to experimentally measured LAD and LCx flow values from a swine at RA and RV pacing. Interpretation of their model results is challenging due to concurrent changes in perfusion pressure and myocardial contractility, in addition to time delay, all of which have a profound impact on myocardial oxygen demand and, hence, on resting flow as well as on hyperemic flow.

Incorporating the mechanism of regional coronary autoregulation to varying myocardial oxygen demand in the present study allowed us to study its impact on myocardial perfusion and flow reserve in isolation, thereby confirming the fundamental role of coronary flow regulation in preserving myocardial perfusion.

### Septal Hypoperfusion in LBBB Is Primarily Caused by Physiological Autoregulatory Response to Reduced Myocardial Oxygen Demand

The underlying mechanism for relative septal hypoperfusion has been the topic of debate for many years. Specifically, it is unclear whether reduced septal perfusion in LBBB represents the pathophysiological restriction of flow due to abnormal asynchronous contraction or the physiological autoregulatory response to altered myocardial oxygen demand (12, 13).

In the present study, we showed that LBBB induces a decrease in diastolic time period and a slight increase in mean intramyocardial pressure, thereby leaving less time for perfusion while compression of the intramyocardial vessels throughout the cardiac cycle is enhanced. Although both factors may play a role in restricting septal flow, their contributions are small at resting heart rate (70 beats/min) and are compensated by coronary autoregulation. Our simulations thereby corroborate an experimental study demonstrating that reduced septal blood flow after onset of LBBB was not accompanied by septal hibernation or lactate production (3), thereby indicating a balanced coupling of myocardial oxygen demand and supply.

It is a well-known phenomenon that patients with LBBB exhibit a heterogeneous pattern of myocardial work, with the early activated septum not contributing much to LV contraction or even wasting work due to systolic stretching while the late-activated lateral wall bears most of the load (1, 3). Correspondingly, several positron emission tomography (PET) studies in patients with LBBB have consistently shown a reduced septal and increased lateral wall metabolism (9–11, 47). A linear correlation between regional myocardial work, metabolism, and blood flow in subjects with and without mechanical dyssynchrony was found in a study by Degtiarova et al. (48). Our model predictions of myocardial oxygen demand and supply in LBBB are in line with these observations, demonstrating pronounced heterogeneity in both factors due to coronary flow regulation to varying demand (Fig. 4). Hence, these findings suggest that relative septal hypoperfusion in LBBB represents a physiological autoregulatory response to a reduction in myocardial oxygen demand.

### Structural Remodeling in LBBB Homogenizes the Distribution of Myocardial Blood Flow per Tissue Weight

The present study demonstrates a redistribution of myocardial blood flow in response to varying myocardial oxygen demand with asynchronous ventricular activation. Similar patterns of myocardial blood flow distribution have been observed with RV pacing and LBBB. Typically, both seem to induce a decrease in septal and an increase in lateral myocardial blood flow (9–13). A reversed distribution pattern of myocardial blood flow across the LV has been observed with LV pacing (6), emphasizing the redistribution of myocardial blood flow away from early activated regions toward late-activated regions.

Although reduced septal perfusion has been frequently observed, the degree of reduction may vary between studies. A possible explanation could be attributed to different imaging modalities, radiopharmaceuticals, study protocols, degrees of dyssynchrony and of remodeling, comorbidities, and small patient populations studied. Although single photon emission computed tomography (SPECT) studies have rather consistently shown relative septal hypoperfusion compared with the lateral wall (11–13), this could be the result of the so-called partial-volume effect caused by the limited resolution in the thinned septum (49). PET has, therefore, emerged as an important imaging technique due to its ability to quantify myocardial blood flow per tissue weight (mL/min/g). However, reports on resting septal perfusion in patients with LBBB with PET imaging are contradictory, ranging from diminished to relatively normal perfusion (9, 10, 14–17).

Our simulations indicate that the degree of structural remodeling is one potential explanation for the inconsistency in observed myocardial blood flow distribution. We showed that the heterogeneous distribution of myocardial blood flow per tissue weight with acute LBBB homogenizes with chronic LBBB (Fig. 4). This is in line with an experimental study by van Oosterhout et al. (6), demonstrating homogenization of myocardial blood flow per tissue weight after long-term ventricular pacing. The large asymmetrical hypertrophy found in their study matched the regional differences in myocardial work and blood flow (6). Such matching, however, may not be perfect (3), explaining observations of septal hypoperfusion in patients with LBBB. Therefore, the degree of structural remodeling associated with LBBB might be of great importance in interpreting PET and SPECT myocardial perfusion images.

### Changes in Myocardial Mass Affect Hyperemic Flow

Although there is an abundance of research on resting myocardial blood flow, data regarding the effect of asynchronous ventricular activation on hyperemic flow is scarce. Regional hyperemic flow was found to be unchanged after short-term RV pacing (17, 50, 51), whereas significant lower hyperemic flow values were observed in the septum in patients with LBBB compared with their values after ∼15 wk of pacing therapy (10). Our model independently reproduced these observations; because hyperemic flow represents a state in which coronary flow is decoupled from myocardial oxygen demand, model-predicted hyperemic flow was unaffected by asynchronous ventricular activation in acute LBBB. Furthermore, under the assumption that regional hyperemic flow is dependent on wall mass, the model showed a reduction in septal and increase in lateral hyperemic flow with chronic LBBB.

A normal capillary density with hypertrophy (52) would explain the significant increase in hyperemic flow observed in athletes (53, 54) and patients with aortic valve stenosis (40) compared with controls. Hyperemic response in the latter study was, however, lower in patients with an extensive increase in LV mass compared with patients with a mild increase (mean increase 147% vs. 27% compared with controls, respectively) (40). A possible explanation could be attributed to insufficient vascular hyperplasia to keep capillary density normal (55). Although these findings suggest that changes in wall mass, to a certain extent, indeed affect hyperemic flow, it is to be speculated whether the slow progression of LBBB and the maximal increase in segmental mass of 25% in our study ensure a normal capillary density. Model-predicted hyperemic response in chronic LBBB is otherwise expected to decrease in the LV lateral wall.

### Regional MFR Has Been Shown to Be Influenced by Loading Conditions, Structural Remodeling, and Vascular Resistance

Given aforementioned observations regarding resting and hyperemic blood flow, septal-to-lateral MFR is presumably increased with acute LBBB, yet unchanged with chronic LBBB. However, clinical studies observed septal-to-lateral MFR to be unchanged in patients with (chronic) LBBB compared with after ∼15 wk of pacing therapy (10), as well as in subjects with acute LBBB as induced by short-term RV pacing (17). In contrary, results from other studies with acute RV pacing showed an increase in LAD coronary flow reserve (50), defined as the ratio of mean hyperemic flow velocity to mean baseline flow velocity, and presumed increase in septal MFR (51), thereby corroborating our results.

Our model framework suggests that the underlying mechanism associated with changes in regional MFR during LBBB may partly be explained by (abnormal) loading conditions, structural remodeling, and vascular resistance. Specifically, the model shows that regional MFR increases with decreases in absolute regional myocardial oxygen demand, which decreases resting myocardial perfusion, as well as with increases in wall thickness, which increases hyperemic perfusion. The opposite holds true for decreases in regional MFR. In addition, the model predicted that MFR decreases toward the apex (i.e., with increase in vascular resistance due to the increase in vascular length to these regions). This was shown in particular by the lower MFR in the distal PDA territory (posterior-septal) compared with the LAD territory (anterior-septal), even though loading conditions and structural remodeling were identical.

### Limitations and Future Work

The observed increase in septal MFR in our study may appear contradictory to the clinical phenomenon of stress perfusion defects often observed in patients with LBBB with normal coronary arteries (17, 56, 57). However, it is important to consider that our modeling approach assumed healthy myocardial tissue and that factors such as heart failure with reduced ejection fraction and coronary microvascular dysfunction could significantly alter the flow reserve and contribute to the observed discrepancy. In addition, structural remodeling in chronic LBBB was mimicked by changing ventricular wall mass while discarding potentially associated changes in ventricular dilatation and stiffness. These aspects should be further investigated in a future study to provide a more comprehensive understanding of the underlying mechanisms responsible for the stress perfusion defects observed in clinical settings. Nevertheless, we have shown that the model predictions align with clinical observations in reproducing heterogeneity in myocardial oxygen demand and perfusion with acute LBBB, as well as homogenization of these factors with chronic LBBB.

Although our study focused on regional coronary flow, it is important to note that the effect of asynchronous ventricular activation on transmural flow was not investigated. We refrained from doing so because the simplified description of myocardial contraction with the one-fiber model does not account for transmural propagation of activation. Consequently, the endo-to-epi flow ratio was held constant at 1.11 in all simulations. To address this limitation, future studies could incorporate a more detailed estimation of oxygen demand, e.g., with a finite-element model of the heart, for a more comprehensive understanding of transmural flow dynamics.

As the mechanisms by which the contracting myocardium affects the coronary flow remain incompletely understood, the intramyocardial pressure was assumed to consist of two components (CEP and VE) to account for major flow features (18), and the ratio of VE to myocardial stiffness (γ) was kept constant in all cases.

As there is limited data available on ventricular oxygen extraction rate, it was assumed constant for both ventricles. This assumption may underestimate simulated resting RCA flow, as it may not necessarily hold true for the RV (58).

### Conclusions

In summary, we have presented a powerful modeling framework that integrates the impediment of myocardial perfusion by myocardial contraction, while simulating coronary autoregulation and wall remodeling by coupling of myocardial oxygen demand to oxygen supply and myocardial growth. Model-based findings suggest that *1*) septal hypoperfusion in LBBB is primarily caused by a physiological autoregulatory response to a reduction in demand, instead of a pathophysiological restriction to coronary flow; *2*) asymmetric hypertrophy, following chronic asynchronous activation, leads to homogenization of myocardial perfusion and flow reserve; *3*) the inconsistency in observed myocardial perfusion and flow reserve with asynchronous ventricular activation can primarily be explained by the degree of dyssynchrony, the degree of regional remodeling of wall mass, and the imaging modality used.

## DATA AVAILABILITY

Source code for this study is openly available at https://doi.org/10.5281/zenodo.8113501.

## SUPPLEMENTAL DATA

10.5281/zenodo.8113501 Supplemental Material: https://doi.org/10.5281/zenodo.8113501.

## GRANTS

J.L. was funded through the Netherlands Organisation for Scientific Research (NWO-ZonMw, VIDI Grant No. 016.176.340) and the Dutch Heart Foundation (Grant No. 2015T082).

## DISCLOSURES

No conflicts of interest, financial or otherwise, are declared by the authors.

## AUTHOR CONTRIBUTIONS

A.G.M., J.L., T.A., F.W.P., and T.D. conceived and designed research; A.G.M. performed experiments; A.G.M. analyzed data; A.G.M., J.L., T.A., F.W.P., and T.D. interpreted results of experiments; A.G.M. prepared figures; A.G.M. drafted manuscript; A.G.M., J.L., T.A., F.W.P., and T.D. edited and revised manuscript; A.G.M., J.L., T.A., F.W.P., and T.D. approved final version of manuscript.
